# Bis(adamantan-1-aminium) hydrogen phosphate fumaric acid sesquisolvate

**DOI:** 10.1107/S1600536812032734

**Published:** 2012-07-25

**Authors:** Mohamed Lahbib Mrad, Matthias Zeller, Kristen J. Hernandez, Mohamed Rzaigui, Cherif Ben Nasr

**Affiliations:** a Laboratoire de Chimie des Matériaux, Faculté des Sciences de Bizerte, 7021 Zarzouna, Tunisia; b Youngstown State University, Department of Chemistry, One University Plaza, Youngstown, Ohio 44555-3663, USA

## Abstract

The asymmetric unit of the title compound, 2C_10_H_18_N^+^·HPO_4_^2−^·1.5C_4_H_4_O_4_, contains two adamantan-1-aminium cations, one hydrogen phosphate anion, and one and a half mol­ecules of fumaric acid, one of which exhibits crystallographic inversion symmetry. Each HPO_4_^2−^ anion is hydrogen bonded, *via* all of its O atoms, to four NH_3_^+^ groups of the adamantan-1-aminium cations, forming chains along [100]. These chains are, in turn, inter­connected *via* a set of O—H⋯O hydrogen bonds involving the fumaric acid solvent mol­ecules, forming layers parallel to (001). Weak C—H⋯O inter­actions lead to a consolidation of the three-dimensional set-up.

## Related literature

For common applications of organic phosphate complexes, see: Coombs *et al.* (1997 ▶); Gani & Wilkie (1995 ▶); Masse *et al.* (1993 ▶); Oliver *et al.* (1995 ▶); Wang *et al.* (1996 ▶). For details of graph-set motifs and theory, see: Bernstein *et al.* (1995 ▶). For reference structural data, see: Kaabi *et al.* (2004 ▶); Chtioui & Jouini (2006 ▶).
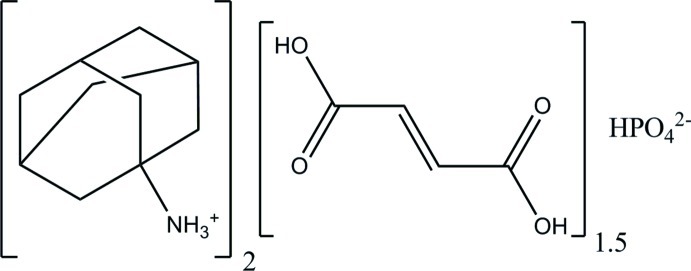


## Experimental

### 

#### Crystal data

2C_10_H_18_N^+^·HO_4_P^2−^·1.5C_4_H_4_O_4_
*M* *_r_* = 574.59
Monoclinic, 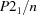

*a* = 12.7555 (16) Å
*b* = 11.1850 (14) Å
*c* = 20.251 (2) Å
β = 105.795 (2)°
*V* = 2780.1 (6) Å^3^
*Z* = 4
Mo *K*α radiation
μ = 0.16 mm^−1^
*T* = 100 K
0.45 × 0.35 × 0.25 mm

#### Data collection

Bruker SMART APEX CCD diffractometer
Absorption correction: multi-scan (*SADABS*; Bruker, 2011 ▶) *T*_min_ = 0.680, *T*_max_ = 0.746
24141 measured reflections
9001 independent reflections
7424 reflections with *I* > 2σ(*I*)
*R*_int_ = 0.028

#### Refinement

*R*[*F* ^2^ > 2σ(*F* ^2^)] = 0.042
*wR*(*F* ^2^) = 0.113
*S* = 1.02
9001 reflections
366 parameters
H atoms treated by a mixture of independent and constrained refinement
Δρ_max_ = 0.51 e Å^−3^
Δρ_min_ = −0.39 e Å^−3^

### 

Data collection: *APEX2* (Bruker, 2011 ▶); cell refinement: *SAINT* (Bruker, 2011 ▶); data reduction: *SAINT*; program(s) used to solve structure: *SHELXTL* (Sheldrick, 2008 ▶); program(s) used to refine structure: *SHELXLE* (Hübschle *et al.*, 2011 ▶); molecular graphics: *SHELXTL*; software used to prepare material for publication: *publCIF* (Westrip, 2010 ▶).

## Supplementary Material

Crystal structure: contains datablock(s) global, I. DOI: 10.1107/S1600536812032734/wm2659sup1.cif

Structure factors: contains datablock(s) I. DOI: 10.1107/S1600536812032734/wm2659Isup2.hkl

Supplementary material file. DOI: 10.1107/S1600536812032734/wm2659Isup3.cml

Additional supplementary materials:  crystallographic information; 3D view; checkCIF report

## Figures and Tables

**Table 1:** Hydrogen-bond geometry (Å, °)

*D*—H⋯*A*	*D*—H	H⋯*A*	*D*⋯*A*	*D*—H⋯*A*
O1—H1⋯O7^i^	0.902 (16)	1.667 (16)	2.5665 (12)	175.0 (15)
O5—H5⋯O2	1.013 (17)	1.486 (17)	2.4918 (12)	171.1 (16)
O8—H8⋯O3^ii^	1.073 (18)	1.396 (18)	2.4659 (12)	174.6 (17)
O10—H10⋯O4	0.955 (18)	1.595 (18)	2.5407 (12)	170.0 (16)
N1*A*—H1*AA*⋯O6^iii^	0.91	1.93	2.8242 (13)	168
N1*A*—H1*AB*⋯O2^iii^	0.91	2.64	3.1486 (12)	117
N1*A*—H1*AC*⋯O2	0.91	1.87	2.7821 (13)	175
N1*B*—H1*BA*⋯O3^iv^	0.91	1.91	2.8201 (13)	174
N1*B*—H1*BB*⋯O4	0.91	1.89	2.8046 (13)	179
N1*B*—H1*BC*⋯O9^iv^	0.91	1.99	2.9016 (13)	177
C3—H3⋯O5	0.95	2.41	2.7379 (14)	100
C6—H6⋯O10^v^	0.95	2.44	2.7730 (15)	100
C7*B*—H7*BA*⋯O7^vi^	0.99	2.50	3.4742 (16)	167

Symmetry codes: (i) 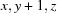; (ii) 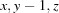; (iii) 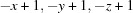; (iv) 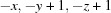; (v) 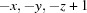; (vi) 
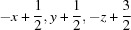
.

**Fig. 1.**
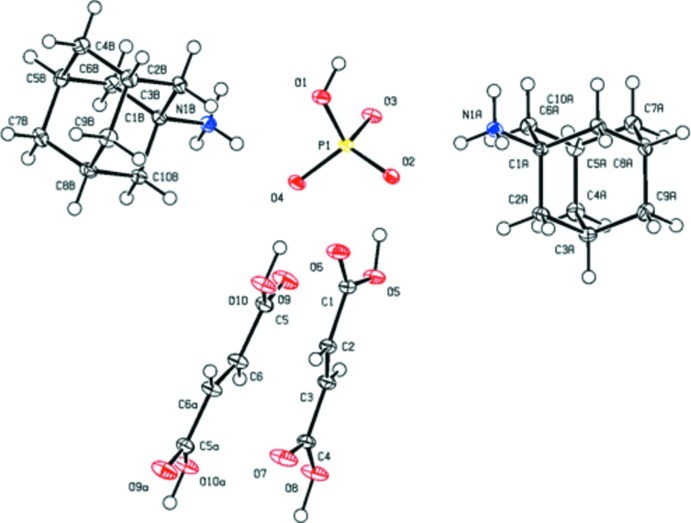
The molecular components of compound (I), showing 50% probability displacement ellipsoids and spheres of arbitrary radius for the H atoms.

**Fig. 2.**
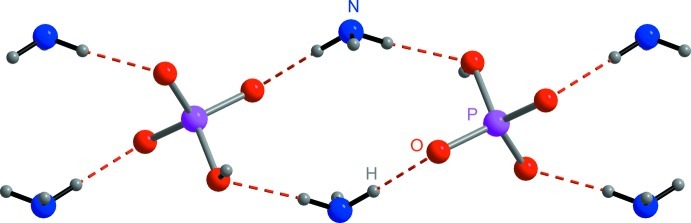
Projection along [100] showing an inorganic chain in the structure of compound (I) with N—H···O hydrogen bonding interactions as broken lines.

**Fig. 3.**
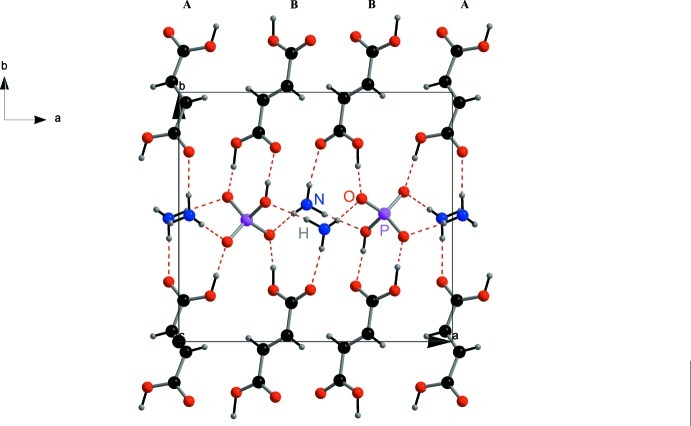
Projection along [001] of a layer in the structure of compound (I). Hydrogen bonds are denoted as broken lines.

**Fig. 4.**
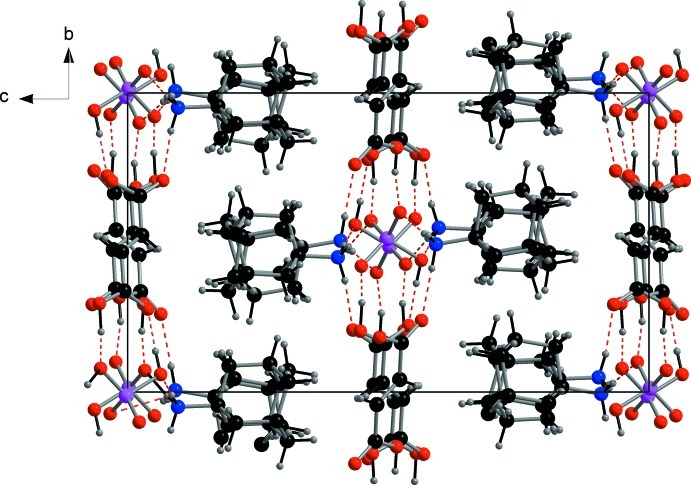
Projection of the structure along [100]. Hydrogen bonds are denoted as dotted lines.

## supplementary crystallographic information

### Comment

Organic phosphate complexes have been widely studied due to their numerous practical and potential uses in various fields in biomolecular sciences, catalysis, fuel cell research, liquid crystal-material development and quadratic non-linear optics studies (Coombs *et al.*, 1997; Gani & Wilkie, 1995; Masse *et al.*, 1993; Oliver *et al.*, 1995; Wang *et al.*, 1996). Here, we report the synthesis and the crystal structure of a new hydrogen phosphate with an organic cation, (C_10_H_18_N)_2_^+^^.^HPO_4_^-^^.^1.5(C_4_H_4_O_4_), (I), formed by the reaction of adamantan-1-aminium fumarate with orthophosphoric acid.

Reaction of the starting materials led to a partial proton transfer from the phosphoric acid to the fumarate anions, which are found to be fully protonated in the structure of compound (I). The asymmetric unit of compound (I) contains one hydrogen phosphate anion, two adamantan-1-aminium cations and one and a half molecules of fumaric acid (Fig. 1), one of which is located on a crystallographic inversion center. Arrangement of the ions and molecules in the crystal structure is governed by a series of strong O—H···O and one N—H···O hydrogen bonds, augmented by a few C—H···O interactions (Table 1, Figures 2–4). In the structure, each HPO_4_^2-^ anion is hydrogen-bonded through all of its oxygen atoms to four NH_3_^+^ groups of the adamantan-1-aminium cations to build infinite hydrogen bonded chains along [100] with an *R*_4_^4^(12) graph set motif (Bernstein *et al.*, 1995) (Fig. 2). These chains are in turn interconnected *via* hydrogen bonds involving the fumaric acid molecules, with two distinct types of hydrogen bonding patterns. For the centrosymmetric fumaric acid molecule both carboxyl groups undergo one O—H···O and one N—H···O hydrogen bond. With respect to the O—H···O bonds, the furmaric acid is the H-bond donor and a phosphate O atom is the corresponding acceptor. With respect to the N—H···O bonds, the H donor is an adamantyl ammonium group and the corresponding acceptor the not-protonated fumaric acid oxygen atom. For the second fumaric acid molecule (located on a general position), the situation is different. One of the two carboxyl groups has a hydrogen bonding pattern similar to that of the centrosymmetric molecule. The second carboxyl group, on the other hand, undergoes two O—H···O hydrogen bonds with two oxygen atoms of the same phosphate anion, with an *R*_2_^2^(8) graph set motif. While the two types of fumaric acid molecules are thus clearly distinct, their roles in the construction of the crystal structure are similar.

The two types of fumaric acid molecules are arranged in skewed stacks where they alternate with one another in an *ABBA* pattern, with *A* being the centrosymmetric fumaric acid molecule (Fig. 3). The stacks extend along [100], in-between the chains of adamantan-1-aminium cations and monohydrogenophosphate anions. Pairs of *B* molecules in the *ABBA* pattern show π–π stacking interactions with an interplanar spacing of 3.108 Å, and a centroid-to-centroid distance of 4.144 Å, indicating substantial slippage of the molecules against one another. Neighboring *A* and *B* molecules are not parallel; the molecular planes are tilted by *ca* 10.8° against one another. Their centroid-to-centroid distance is 4.306 Å, and actual close contacts are limited to some O···O interactions. *A* and *B* molecules are not π—π-stacked with one another.

Through their O—H···O and N—H···O hydrogen bonds to the hydrogen phosphate anions and adamantan-1-aminium cations, the fumaric acid molecules give rise to two-dimensional infinite layers parallel to (001) (Fig. 3). Fig. 4 shows that the adamantan-1-aminium cation and monohydrogenophosphate anion chains extend along [100] at (*x*, 0, 0) and (*x*, 1/2, 1/2), while the layers cross the unit cell at *c* = *n*/2. The adamantan-1-aminium cations of parallel layers interdigitate with one another and are located in alternate pairs on either side of the layers, leading to an extended three-dimensional structure (Fig. 4), which is further consolidated by a small number of weak C—H···O interactions (Table 1).

The detailed geometry of the HPO_4_^2-^ group shows two kinds of P—O distances. The shorter ones (1.5252 (9), 1.5330 (8) and 1.5148 (8) Å correspond to the non-protonated oxygen atoms, while the largest one (1.5946 (8) Å) is associated with the P—OH bond. This is in agreement with literature data for the monohydrogenophosphate anion in similar arrangements (*e.g.* Chtioui & Jouini, 2006; Kaabi *et al.*, 2004). The O—H···O interactions show elongated O—H distances typical for very strong hydrogen bonds and range from 0.902 (16) for H1 to 1.073 (18) for H8 (Table 1).

### Experimental

Crystals of the title compound were prepared at room temperature by slow addition of a solution of orthophosphoric acid (3 mmol in 20 ml of water) to an alcoholic solution of adamantan-1-aminium fumarate (6 mmol in 20 ml of ethanol). The acid was added until the alcoholic solution became turbid. After filtration, the solution was allowed to slowly evaporate at room temperature over several days leading to formation of transparent prismatic crystals with suitable dimensions for single-crystal structural analysis (yield 55%). The crystals are stable for months under normal conditions of temperature and humidity.

### Refinement

C, and N bound H atoms were placed in calculated positions riding on their respective carrier atom with C—H in the range 0.95–1.00 and N—H of 0.91 Å. Ammonium H atoms were allowed to rotate but not to tip to best fit the observed electron density distribution. *U*_iso_(H) values were constrained to be in the range of 1.2 *U*_eq_ of the parent atom for C bound H atoms, and 1.5 times *U*_eq_ for N and O bound H atoms. O bound H atoms were located in difference density Fourier maps, and their positons were freely refined.

### Crystal data

**Table:**

2C_10_H_18_N^+^·HO_4_P^2^^−^·1.5C_4_H_4_O_4_	*F*(000) = 1232
*M**_r_* = 574.59	*D*_x_ = 1.373 Mg m^−^^3^
Monoclinic, *P*2_1_/*n*	Mo *K*α radiation, λ = 0.71073 Å
Hall symbol: -P 2yn	Cell parameters from 6149 reflections
*a* = 12.7555 (16) Å	θ = 2.5–31.5°
*b* = 11.1850 (14) Å	µ = 0.16 mm^−^^1^
*c* = 20.251 (2) Å	*T* = 100 K
β = 105.795 (2)°	Block, colourless
*V* = 2780.1 (6) Å^3^	0.45 × 0.35 × 0.25 mm
*Z* = 4	

### Data collection

**Table:**

Bruker SMART APEX CCD diffractometer	9001 independent reflections
Radiation source: fine-focus sealed tube	7424 reflections with *I* > 2σ(*I*)
Graphite monochromator	*R*_int_ = 0.028
ω scans	θ_max_ = 32.2°, θ_min_ = 1.7°
Absorption correction: multi-scan (*SADABS*; Bruker, 2011)	*h* = −18→18
*T*_min_ = 0.680, *T*_max_ = 0.746	*k* = −16→16
24141 measured reflections	*l* = −27→28

### Refinement

**Table:**

Refinement on *F*^2^	Primary atom site location: structure-invariant direct methods
Least-squares matrix: full	Secondary atom site location: difference Fourier map
*R*[*F*^2^ > 2σ(*F*^2^)] = 0.042	Hydrogen site location: inferred from neighbouring sites
*wR*(*F*^2^) = 0.113	H atoms treated by a mixture of independent and constrained refinement
*S* = 1.02	*w* = 1/[σ^2^(*F*_o_^2^) + (0.0581*P*)^2^ + 0.684*P*] where *P* = (*F*_o_^2^ + 2*F*_c_^2^)/3
9001 reflections	(Δ/σ)_max_ = 0.001
366 parameters	Δρ_max_ = 0.51 e Å^−^^3^
0 restraints	Δρ_min_ = −0.39 e Å^−^^3^

### Special details

**Table:**

Geometry. All e.s.d.'s (except the e.s.d. in the dihedral angle between two l.s. planes) are estimated using the full covariance matrix. The cell e.s.d.'s are taken into account individually in the estimation of e.s.d.'s in distances, angles and torsion angles; correlations between e.s.d.'s in cell parameters are only used when they are defined by crystal symmetry. An approximate (isotropic) treatment of cell e.s.d.'s is used for estimating e.s.d.'s involving l.s. planes.
Refinement. Refinement of *F*^2^ against ALL reflections. The weighted *R*-factor *wR* and goodness of fit *S* are based on *F*^2^, conventional *R*-factors *R* are based on *F*, with *F* set to zero for negative *F*^2^. The threshold expression of *F*^2^ > σ(*F*^2^) is used only for calculating *R*-factors(gt) *etc*. and is not relevant to the choice of reflections for refinement. *R*-factors based on *F*^2^ are statistically about twice as large as those based on *F*, and *R*- factors based on ALL data will be even larger.

### Fractional atomic coordinates and isotropic or equivalent isotropic displacement parameters (Å^2^)

**Table:**

	*x*	*y*	*z*	*U*_iso_*/*U*_eq_	
C1	0.41137 (10)	0.15548 (10)	0.52298 (6)	0.0156 (2)	
C2	0.39579 (10)	0.02404 (10)	0.52724 (6)	0.0161 (2)	
H2	0.4500	−0.0216	0.5586	0.019*	
C3	0.30842 (10)	−0.03144 (10)	0.48851 (6)	0.0171 (2)	
H3	0.2547	0.0141	0.4567	0.020*	
C4	0.29194 (10)	−0.16227 (10)	0.49331 (6)	0.0166 (2)	
O5	0.34561 (7)	0.20545 (7)	0.46935 (4)	0.01976 (18)	
H5	0.3451 (13)	0.2959 (15)	0.4725 (8)	0.030*	
O6	0.48114 (8)	0.20734 (7)	0.56762 (5)	0.0228 (2)	
O7	0.35313 (8)	−0.22223 (8)	0.53892 (5)	0.0284 (2)	
O8	0.21020 (8)	−0.20382 (8)	0.44631 (5)	0.0254 (2)	
H8	0.2001 (15)	−0.2987 (16)	0.4495 (9)	0.038*	
C5	0.01715 (10)	0.16473 (10)	0.47501 (6)	0.0168 (2)	
C6	−0.02584 (10)	0.04172 (10)	0.47816 (6)	0.0182 (2)	
H6	−0.0940	0.0217	0.4472	0.022*	
O9	−0.03696 (8)	0.23937 (8)	0.43612 (5)	0.0260 (2)	
O10	0.11474 (7)	0.18302 (8)	0.51613 (5)	0.0228 (2)	
H10	0.1367 (14)	0.2640 (16)	0.5132 (9)	0.034*	
N1A	0.46741 (8)	0.54933 (8)	0.40323 (5)	0.01350 (18)	
H1AA	0.4775	0.6270	0.4168	0.020*	
H1AB	0.5325	0.5103	0.4153	0.020*	
H1AC	0.4205	0.5138	0.4239	0.020*	
C1A	0.42108 (8)	0.54404 (9)	0.32689 (5)	0.01162 (19)	
C2A	0.40449 (9)	0.41239 (10)	0.30542 (6)	0.0147 (2)	
H2AA	0.3540	0.3740	0.3285	0.018*	
H2AB	0.4750	0.3695	0.3192	0.018*	
C3A	0.35690 (9)	0.40584 (10)	0.22711 (6)	0.0159 (2)	
H3A	0.3457	0.3203	0.2124	0.019*	
C4A	0.24735 (9)	0.47186 (11)	0.20731 (6)	0.0183 (2)	
H4AA	0.1966	0.4340	0.2303	0.022*	
H4AB	0.2148	0.4665	0.1571	0.022*	
C5A	0.26430 (9)	0.60354 (10)	0.22878 (6)	0.0166 (2)	
H5A	0.1928	0.6463	0.2153	0.020*	
C6A	0.31220 (9)	0.61047 (10)	0.30720 (6)	0.0151 (2)	
H6AA	0.3233	0.6951	0.3218	0.018*	
H6AB	0.2612	0.5736	0.3304	0.018*	
C7A	0.34354 (10)	0.66261 (10)	0.19371 (6)	0.0174 (2)	
H7AA	0.3543	0.7475	0.2078	0.021*	
H7AB	0.3128	0.6597	0.1433	0.021*	
C8A	0.45331 (9)	0.59670 (10)	0.21408 (6)	0.0151 (2)	
H8A	0.5049	0.6351	0.1911	0.018*	
C9A	0.43597 (9)	0.46543 (10)	0.19196 (6)	0.0168 (2)	
H9AA	0.5066	0.4228	0.2047	0.020*	
H9AB	0.4059	0.4607	0.1415	0.020*	
C10A	0.50088 (9)	0.60313 (10)	0.29247 (6)	0.0142 (2)	
H10A	0.5719	0.5613	0.3061	0.017*	
H10B	0.5127	0.6876	0.3072	0.017*	
N1B	0.03808 (8)	0.50606 (8)	0.59162 (5)	0.01363 (18)	
H1BA	−0.0303	0.4753	0.5764	0.020*	
H1BB	0.0825	0.4704	0.5691	0.020*	
H1BC	0.0360	0.5862	0.5836	0.020*	
C1B	0.08106 (9)	0.48352 (9)	0.66724 (5)	0.01162 (19)	
C2B	0.19608 (9)	0.53554 (10)	0.69246 (6)	0.0147 (2)	
H2BA	0.1943	0.6226	0.6834	0.018*	
H2BB	0.2444	0.4976	0.6677	0.018*	
C3B	0.23979 (9)	0.51206 (10)	0.76999 (6)	0.0158 (2)	
H3B	0.3151	0.5455	0.7869	0.019*	
C4B	0.16533 (10)	0.57196 (10)	0.80815 (6)	0.0175 (2)	
H4BA	0.1640	0.6594	0.8004	0.021*	
H4BB	0.1936	0.5572	0.8580	0.021*	
C5B	0.04941 (9)	0.52090 (10)	0.78197 (6)	0.0161 (2)	
H5B	0.0006	0.5598	0.8068	0.019*	
C6B	0.00607 (9)	0.54468 (10)	0.70445 (6)	0.0150 (2)	
H6BA	−0.0689	0.5129	0.6873	0.018*	
H6BB	0.0040	0.6318	0.6956	0.018*	
C7B	0.05222 (10)	0.38520 (10)	0.79445 (6)	0.0174 (2)	
H7BA	0.0788	0.3688	0.8443	0.021*	
H7BB	−0.0222	0.3520	0.7776	0.021*	
C8B	0.12762 (9)	0.32495 (10)	0.75694 (6)	0.0157 (2)	
H8B	0.1296	0.2369	0.7657	0.019*	
C9B	0.24281 (9)	0.37652 (10)	0.78298 (6)	0.0174 (2)	
H9BA	0.2916	0.3377	0.7589	0.021*	
H9BB	0.2717	0.3605	0.8327	0.021*	
C10B	0.08390 (9)	0.34840 (9)	0.67946 (6)	0.0145 (2)	
H10C	0.1317	0.3096	0.6546	0.017*	
H10D	0.0097	0.3146	0.6622	0.017*	
O1	0.31637 (7)	0.55891 (7)	0.56349 (4)	0.01518 (16)	
H1	0.3252 (13)	0.6363 (14)	0.5537 (8)	0.023*	
O2	0.33124 (7)	0.42767 (7)	0.46675 (4)	0.01513 (16)	
O3	0.17816 (6)	0.57897 (7)	0.44840 (4)	0.01517 (16)	
O4	0.17691 (7)	0.39954 (7)	0.52248 (4)	0.01583 (16)	
P1	0.24805 (2)	0.48755 (2)	0.497620 (14)	0.01113 (7)	

### Atomic displacement parameters (Å^2^)

**Table:**

	*U*^11^	*U*^22^	*U*^33^	*U*^12^	*U*^13^	*U*^23^
C1	0.0193 (5)	0.0112 (4)	0.0168 (5)	−0.0023 (4)	0.0059 (4)	−0.0016 (4)
C2	0.0224 (6)	0.0105 (4)	0.0156 (5)	−0.0006 (4)	0.0057 (4)	0.0005 (4)
C3	0.0234 (6)	0.0105 (4)	0.0166 (5)	−0.0019 (4)	0.0043 (4)	0.0007 (4)
C4	0.0219 (6)	0.0107 (4)	0.0162 (5)	−0.0037 (4)	0.0036 (4)	−0.0006 (4)
O5	0.0249 (4)	0.0106 (4)	0.0205 (4)	−0.0016 (3)	0.0006 (3)	0.0001 (3)
O6	0.0289 (5)	0.0134 (4)	0.0219 (4)	−0.0050 (3)	0.0000 (4)	−0.0023 (3)
O7	0.0360 (5)	0.0137 (4)	0.0252 (5)	−0.0072 (4)	−0.0093 (4)	0.0042 (3)
O8	0.0279 (5)	0.0117 (4)	0.0279 (5)	−0.0054 (3)	−0.0072 (4)	0.0030 (3)
C5	0.0189 (5)	0.0123 (5)	0.0199 (5)	−0.0022 (4)	0.0062 (4)	0.0007 (4)
C6	0.0186 (5)	0.0117 (5)	0.0234 (6)	−0.0034 (4)	0.0043 (4)	0.0005 (4)
O9	0.0270 (5)	0.0151 (4)	0.0299 (5)	−0.0032 (3)	−0.0024 (4)	0.0068 (3)
O10	0.0200 (4)	0.0124 (4)	0.0322 (5)	−0.0042 (3)	0.0004 (4)	0.0041 (3)
N1A	0.0132 (4)	0.0131 (4)	0.0137 (4)	−0.0023 (3)	0.0028 (3)	−0.0004 (3)
C1A	0.0108 (4)	0.0111 (4)	0.0127 (5)	−0.0008 (3)	0.0028 (4)	−0.0007 (4)
C2A	0.0165 (5)	0.0110 (4)	0.0162 (5)	−0.0019 (4)	0.0035 (4)	−0.0002 (4)
C3A	0.0168 (5)	0.0138 (5)	0.0163 (5)	−0.0028 (4)	0.0033 (4)	−0.0030 (4)
C4A	0.0136 (5)	0.0229 (6)	0.0173 (5)	−0.0047 (4)	0.0022 (4)	−0.0016 (4)
C5A	0.0112 (5)	0.0199 (5)	0.0176 (5)	0.0023 (4)	0.0020 (4)	0.0007 (4)
C6A	0.0119 (5)	0.0168 (5)	0.0168 (5)	0.0015 (4)	0.0043 (4)	−0.0007 (4)
C7A	0.0171 (5)	0.0179 (5)	0.0158 (5)	0.0013 (4)	0.0020 (4)	0.0030 (4)
C8A	0.0139 (5)	0.0174 (5)	0.0145 (5)	−0.0024 (4)	0.0049 (4)	0.0018 (4)
C9A	0.0147 (5)	0.0199 (5)	0.0162 (5)	0.0004 (4)	0.0049 (4)	−0.0024 (4)
C10A	0.0113 (5)	0.0152 (5)	0.0158 (5)	−0.0026 (4)	0.0034 (4)	0.0005 (4)
N1B	0.0134 (4)	0.0132 (4)	0.0135 (4)	−0.0001 (3)	0.0024 (3)	0.0012 (3)
C1B	0.0108 (4)	0.0116 (4)	0.0122 (5)	−0.0003 (3)	0.0026 (4)	0.0008 (3)
C2B	0.0124 (5)	0.0160 (5)	0.0161 (5)	−0.0022 (4)	0.0047 (4)	0.0011 (4)
C3B	0.0116 (5)	0.0184 (5)	0.0162 (5)	−0.0024 (4)	0.0017 (4)	−0.0001 (4)
C4B	0.0195 (5)	0.0169 (5)	0.0156 (5)	−0.0026 (4)	0.0041 (4)	−0.0020 (4)
C5B	0.0144 (5)	0.0189 (5)	0.0161 (5)	0.0016 (4)	0.0063 (4)	−0.0003 (4)
C6B	0.0124 (5)	0.0156 (5)	0.0173 (5)	0.0025 (4)	0.0044 (4)	0.0007 (4)
C7B	0.0165 (5)	0.0192 (5)	0.0173 (5)	−0.0035 (4)	0.0058 (4)	0.0033 (4)
C8B	0.0181 (5)	0.0119 (4)	0.0160 (5)	0.0001 (4)	0.0030 (4)	0.0022 (4)
C9B	0.0143 (5)	0.0185 (5)	0.0179 (5)	0.0039 (4)	0.0020 (4)	0.0022 (4)
C10B	0.0161 (5)	0.0109 (4)	0.0163 (5)	0.0001 (4)	0.0040 (4)	0.0006 (4)
O1	0.0176 (4)	0.0111 (3)	0.0147 (4)	−0.0020 (3)	0.0007 (3)	0.0000 (3)
O2	0.0170 (4)	0.0109 (3)	0.0198 (4)	−0.0002 (3)	0.0089 (3)	0.0008 (3)
O3	0.0142 (4)	0.0101 (3)	0.0186 (4)	−0.0009 (3)	0.0000 (3)	0.0011 (3)
O4	0.0163 (4)	0.0115 (3)	0.0215 (4)	−0.0036 (3)	0.0083 (3)	−0.0003 (3)
P1	0.01134 (13)	0.00869 (12)	0.01317 (13)	−0.00120 (9)	0.00301 (10)	0.00048 (9)

### Geometric parameters (Å, º)

**Table:**

C1—O6	1.2280 (13)	C8A—C10A	1.5392 (15)
C1—O5	1.3036 (14)	C8A—H8A	1.0000
C1—C2	1.4891 (15)	C9A—H9AA	0.9900
C2—C3	1.3291 (16)	C9A—H9AB	0.9900
C2—H2	0.9500	C10A—H10A	0.9900
C3—C4	1.4853 (15)	C10A—H10B	0.9900
C3—H3	0.9500	N1B—C1B	1.5006 (13)
C4—O7	1.2328 (14)	N1B—H1BA	0.9100
C4—O8	1.2918 (13)	N1B—H1BB	0.9100
O5—H5	1.013 (17)	N1B—H1BC	0.9100
O8—H8	1.073 (18)	C1B—C10B	1.5303 (15)
C5—O9	1.2237 (14)	C1B—C6B	1.5307 (16)
C5—O10	1.3117 (14)	C1B—C2B	1.5310 (15)
C5—C6	1.4889 (16)	C2B—C3B	1.5390 (15)
C6—C6^i^	1.330 (2)	C2B—H2BA	0.9900
C6—H6	0.9500	C2B—H2BB	0.9900
O10—H10	0.955 (18)	C3B—C4B	1.5322 (17)
N1A—C1A	1.4984 (13)	C3B—C9B	1.5375 (16)
N1A—H1AA	0.9100	C3B—H3B	1.0000
N1A—H1AB	0.9100	C4B—C5B	1.5384 (16)
N1A—H1AC	0.9100	C4B—H4BA	0.9900
C1A—C6A	1.5291 (15)	C4B—H4BB	0.9900
C1A—C10A	1.5314 (16)	C5B—C7B	1.5376 (16)
C1A—C2A	1.5337 (15)	C5B—C6B	1.5390 (16)
C2A—C3A	1.5379 (15)	C5B—H5B	1.0000
C2A—H2AA	0.9900	C6B—H6BA	0.9900
C2A—H2AB	0.9900	C6B—H6BB	0.9900
C3A—C4A	1.5341 (17)	C7B—C8B	1.5341 (17)
C3A—C9A	1.5359 (17)	C7B—H7BA	0.9900
C3A—H3A	1.0000	C7B—H7BB	0.9900
C4A—C5A	1.5343 (17)	C8B—C9B	1.5322 (16)
C4A—H4AA	0.9900	C8B—C10B	1.5379 (15)
C4A—H4AB	0.9900	C8B—H8B	1.0000
C5A—C7A	1.5342 (17)	C9B—H9BA	0.9900
C5A—C6A	1.5405 (16)	C9B—H9BB	0.9900
C5A—H5A	1.0000	C10B—H10C	0.9900
C6A—H6AA	0.9900	C10B—H10D	0.9900
C6A—H6AB	0.9900	O1—P1	1.5946 (8)
C7A—C8A	1.5361 (16)	O1—H1	0.902 (16)
C7A—H7AA	0.9900	O2—P1	1.5252 (9)
C7A—H7AB	0.9900	O3—P1	1.5330 (8)
C8A—C9A	1.5335 (16)	O4—P1	1.5148 (8)
			
O6—C1—O5	125.72 (10)	H9AA—C9A—H9AB	108.2
O6—C1—C2	120.33 (10)	C1A—C10A—C8A	108.97 (9)
O5—C1—C2	113.94 (10)	C1A—C10A—H10A	109.9
C3—C2—C1	122.03 (10)	C8A—C10A—H10A	109.9
C3—C2—H2	119.0	C1A—C10A—H10B	109.9
C1—C2—H2	119.0	C8A—C10A—H10B	109.9
C2—C3—C4	122.16 (11)	H10A—C10A—H10B	108.3
C2—C3—H3	118.9	C1B—N1B—H1BA	109.5
C4—C3—H3	118.9	C1B—N1B—H1BB	109.5
O7—C4—O8	125.16 (10)	H1BA—N1B—H1BB	109.5
O7—C4—C3	120.86 (10)	C1B—N1B—H1BC	109.5
O8—C4—C3	113.98 (10)	H1BA—N1B—H1BC	109.5
C1—O5—H5	112.9 (9)	H1BB—N1B—H1BC	109.5
C4—O8—H8	113.4 (9)	N1B—C1B—C10B	108.48 (8)
O9—C5—O10	125.00 (11)	N1B—C1B—C6B	108.88 (8)
O9—C5—C6	120.55 (11)	C10B—C1B—C6B	110.62 (9)
O10—C5—C6	114.45 (10)	N1B—C1B—C2B	109.10 (9)
C6^i^—C6—C5	123.72 (14)	C10B—C1B—C2B	110.06 (9)
C6^i^—C6—H6	118.1	C6B—C1B—C2B	109.66 (9)
C5—C6—H6	118.1	C1B—C2B—C3B	108.88 (9)
C5—O10—H10	110.6 (10)	C1B—C2B—H2BA	109.9
C1A—N1A—H1AA	109.5	C3B—C2B—H2BA	109.9
C1A—N1A—H1AB	109.5	C1B—C2B—H2BB	109.9
H1AA—N1A—H1AB	109.5	C3B—C2B—H2BB	109.9
C1A—N1A—H1AC	109.5	H2BA—C2B—H2BB	108.3
H1AA—N1A—H1AC	109.5	C4B—C3B—C9B	109.57 (10)
H1AB—N1A—H1AC	109.5	C4B—C3B—C2B	109.74 (9)
N1A—C1A—C6A	108.97 (9)	C9B—C3B—C2B	109.15 (9)
N1A—C1A—C10A	109.17 (8)	C4B—C3B—H3B	109.5
C6A—C1A—C10A	109.91 (9)	C9B—C3B—H3B	109.5
N1A—C1A—C2A	108.40 (8)	C2B—C3B—H3B	109.5
C6A—C1A—C2A	110.31 (9)	C3B—C4B—C5B	109.48 (9)
C10A—C1A—C2A	110.05 (9)	C3B—C4B—H4BA	109.8
C1A—C2A—C3A	108.87 (9)	C5B—C4B—H4BA	109.8
C1A—C2A—H2AA	109.9	C3B—C4B—H4BB	109.8
C3A—C2A—H2AA	109.9	C5B—C4B—H4BB	109.8
C1A—C2A—H2AB	109.9	H4BA—C4B—H4BB	108.2
C3A—C2A—H2AB	109.9	C7B—C5B—C4B	109.43 (9)
H2AA—C2A—H2AB	108.3	C7B—C5B—C6B	108.90 (9)
C4A—C3A—C9A	109.65 (9)	C4B—C5B—C6B	109.39 (10)
C4A—C3A—C2A	108.80 (10)	C7B—C5B—H5B	109.7
C9A—C3A—C2A	109.43 (9)	C4B—C5B—H5B	109.7
C4A—C3A—H3A	109.6	C6B—C5B—H5B	109.7
C9A—C3A—H3A	109.6	C1B—C6B—C5B	109.02 (9)
C2A—C3A—H3A	109.6	C1B—C6B—H6BA	109.9
C3A—C4A—C5A	109.88 (9)	C5B—C6B—H6BA	109.9
C3A—C4A—H4AA	109.7	C1B—C6B—H6BB	109.9
C5A—C4A—H4AA	109.7	C5B—C6B—H6BB	109.9
C3A—C4A—H4AB	109.7	H6BA—C6B—H6BB	108.3
C5A—C4A—H4AB	109.7	C8B—C7B—C5B	110.00 (9)
H4AA—C4A—H4AB	108.2	C8B—C7B—H7BA	109.7
C7A—C5A—C4A	109.99 (10)	C5B—C7B—H7BA	109.7
C7A—C5A—C6A	109.22 (9)	C8B—C7B—H7BB	109.7
C4A—C5A—C6A	109.04 (9)	C5B—C7B—H7BB	109.7
C7A—C5A—H5A	109.5	H7BA—C7B—H7BB	108.2
C4A—C5A—H5A	109.5	C9B—C8B—C7B	109.63 (9)
C6A—C5A—H5A	109.5	C9B—C8B—C10B	109.54 (10)
C1A—C6A—C5A	108.76 (9)	C7B—C8B—C10B	109.15 (9)
C1A—C6A—H6AA	109.9	C9B—C8B—H8B	109.5
C5A—C6A—H6AA	109.9	C7B—C8B—H8B	109.5
C1A—C6A—H6AB	109.9	C10B—C8B—H8B	109.5
C5A—C6A—H6AB	109.9	C8B—C9B—C3B	109.59 (9)
H6AA—C6A—H6AB	108.3	C8B—C9B—H9BA	109.8
C5A—C7A—C8A	109.65 (9)	C3B—C9B—H9BA	109.8
C5A—C7A—H7AA	109.7	C8B—C9B—H9BB	109.8
C8A—C7A—H7AA	109.7	C3B—C9B—H9BB	109.8
C5A—C7A—H7AB	109.7	H9BA—C9B—H9BB	108.2
C8A—C7A—H7AB	109.7	C1B—C10B—C8B	108.63 (9)
H7AA—C7A—H7AB	108.2	C1B—C10B—H10C	110.0
C9A—C8A—C7A	109.43 (9)	C8B—C10B—H10C	110.0
C9A—C8A—C10A	109.33 (9)	C1B—C10B—H10D	110.0
C7A—C8A—C10A	109.22 (9)	C8B—C10B—H10D	110.0
C9A—C8A—H8A	109.6	H10C—C10B—H10D	108.3
C7A—C8A—H8A	109.6	P1—O1—H1	111.7 (10)
C10A—C8A—H8A	109.6	O4—P1—O2	113.41 (5)
C8A—C9A—C3A	109.93 (9)	O4—P1—O3	110.77 (5)
C8A—C9A—H9AA	109.7	O2—P1—O3	111.89 (5)
C3A—C9A—H9AA	109.7	O4—P1—O1	106.72 (5)
C8A—C9A—H9AB	109.7	O2—P1—O1	106.25 (5)
C3A—C9A—H9AB	109.7	O3—P1—O1	107.38 (4)
			
O6—C1—C2—C3	166.16 (13)	C2A—C1A—C10A—C8A	60.76 (11)
O5—C1—C2—C3	−13.71 (17)	C9A—C8A—C10A—C1A	−59.81 (12)
C1—C2—C3—C4	−179.17 (11)	C7A—C8A—C10A—C1A	59.90 (11)
C2—C3—C4—O7	8.1 (2)	N1B—C1B—C2B—C3B	−179.94 (9)
C2—C3—C4—O8	−171.71 (12)	C10B—C1B—C2B—C3B	−61.02 (12)
O9—C5—C6—C6^i^	−176.14 (16)	C6B—C1B—C2B—C3B	60.89 (11)
O10—C5—C6—C6^i^	4.2 (2)	C1B—C2B—C3B—C4B	−60.08 (12)
N1A—C1A—C2A—C3A	179.99 (9)	C1B—C2B—C3B—C9B	60.01 (12)
C6A—C1A—C2A—C3A	60.75 (12)	C9B—C3B—C4B—C5B	−60.17 (12)
C10A—C1A—C2A—C3A	−60.69 (11)	C2B—C3B—C4B—C5B	59.67 (12)
C1A—C2A—C3A—C4A	−60.09 (12)	C3B—C4B—C5B—C7B	59.62 (12)
C1A—C2A—C3A—C9A	59.70 (12)	C3B—C4B—C5B—C6B	−59.62 (12)
C9A—C3A—C4A—C5A	−58.77 (12)	N1B—C1B—C6B—C5B	179.56 (9)
C2A—C3A—C4A—C5A	60.88 (12)	C10B—C1B—C6B—C5B	60.44 (11)
C3A—C4A—C5A—C7A	58.94 (12)	C2B—C1B—C6B—C5B	−61.14 (11)
C3A—C4A—C5A—C6A	−60.81 (12)	C7B—C5B—C6B—C1B	−59.38 (12)
N1A—C1A—C6A—C5A	−179.36 (9)	C4B—C5B—C6B—C1B	60.18 (12)
C10A—C1A—C6A—C5A	61.06 (11)	C4B—C5B—C7B—C8B	−59.23 (12)
C2A—C1A—C6A—C5A	−60.47 (12)	C6B—C5B—C7B—C8B	60.30 (12)
C7A—C5A—C6A—C1A	−60.38 (11)	C5B—C7B—C8B—C9B	59.24 (12)
C4A—C5A—C6A—C1A	59.84 (12)	C5B—C7B—C8B—C10B	−60.74 (12)
C4A—C5A—C7A—C8A	−59.30 (12)	C7B—C8B—C9B—C3B	−59.46 (12)
C6A—C5A—C7A—C8A	60.33 (12)	C10B—C8B—C9B—C3B	60.29 (12)
C5A—C7A—C8A—C9A	59.57 (12)	C4B—C3B—C9B—C8B	60.13 (12)
C5A—C7A—C8A—C10A	−60.07 (12)	C2B—C3B—C9B—C8B	−60.06 (12)
C7A—C8A—C9A—C3A	−59.78 (12)	N1B—C1B—C10B—C8B	−179.85 (9)
C10A—C8A—C9A—C3A	59.79 (12)	C6B—C1B—C10B—C8B	−60.49 (11)
C4A—C3A—C9A—C8A	59.42 (12)	C2B—C1B—C10B—C8B	60.85 (12)
C2A—C3A—C9A—C8A	−59.85 (12)	C9B—C8B—C10B—C1B	−60.14 (12)
N1A—C1A—C10A—C8A	179.61 (8)	C7B—C8B—C10B—C1B	59.90 (12)
C6A—C1A—C10A—C8A	−60.93 (11)		

Symmetry code: (i) −*x*, −*y*, −*z*+1.

### Hydrogen-bond geometry (Å, º)

**Table:**

*D*—H···*A*	*D*—H	H···*A*	*D*···*A*	*D*—H···*A*
O1—H1···O7^ii^	0.902 (16)	1.667 (16)	2.5665 (12)	175.0 (15)
O5—H5···O2	1.013 (17)	1.486 (17)	2.4918 (12)	171.1 (16)
O8—H8···O3^iii^	1.073 (18)	1.396 (18)	2.4659 (12)	174.6 (17)
O10—H10···O4	0.955 (18)	1.595 (18)	2.5407 (12)	170.0 (16)
N1*A*—H1*AA*···O6^iv^	0.91	1.93	2.8242 (13)	168
N1*A*—H1*AB*···O2^iv^	0.91	2.64	3.1486 (12)	117
N1*A*—H1*AC*···O2	0.91	1.87	2.7821 (13)	175
N1*B*—H1*BA*···O3^v^	0.91	1.91	2.8201 (13)	174
N1*B*—H1*BB*···O4	0.91	1.89	2.8046 (13)	179
N1*B*—H1*BC*···O9^v^	0.91	1.99	2.9016 (13)	177
C3—H3···O5	0.95	2.41	2.7379 (14)	100
C6—H6···O10^i^	0.95	2.44	2.7730 (15)	100
C7*B*—H7*BA*···O7^vi^	0.99	2.50	3.4742 (16)	167

Symmetry codes: (i) −*x*, −*y*, −*z*+1; (ii) *x*, *y*+1, *z*; (iii) *x*, *y*−1, *z*; (iv) −*x*+1, −*y*+1, −*z*+1; (v) −*x*, −*y*+1, −*z*+1; (vi) −*x*+1/2, *y*+1/2, −*z*+3/2.
